# Carney Triad, Carney-Stratakis Syndrome, 3PAS and Other Tumors Due to SDH Deficiency

**DOI:** 10.3389/fendo.2021.680609

**Published:** 2021-05-03

**Authors:** Georgia Pitsava, Nikolaos Settas, Fabio R. Faucz, Constantine A. Stratakis

**Affiliations:** ^1^Division of Intramural Population Health Research, Eunice Kennedy Shriver National Institutes of Child Health and Human Development, National Institutes of Health, Bethesda, MD, United States; ^2^Section on Endocrinology and Genetics, Eunice Kennedy Shriver National Institute of Child Health and Human Development, National Institutes of Health, Bethesda, MD, United States

**Keywords:** Succinate dehydrogenase (SDH), GIST, paraganglioma, Carney triad, Carney-Stratakis syndrome, SDHB

## Abstract

Succinate dehydrogenase (SDH) is a key respiratory enzyme that links Krebs cycle and electron transport chain and is comprised of four subunits SDHA, SDHB, SDHC and SDHD. All *SDH*-deficient tumors are caused by or secondary to loss of SDH activity. As many as half of the familial cases of paragangliomas (PGLs) and pheochromocytomas (PHEOs) are due to mutations of the *SDHx* subunits. Gastrointestinal stromal tumors (GISTs) associated with *SDH* deficiency are negative for *KIT/PDGFRA* mutations and present with distinctive clinical features such as early onset (usually childhood or adolescence) and almost exclusively gastric location. *SDH*-deficient GISTs may be part of distinct clinical syndromes, Carney-Stratakis syndrome (CSS) or dyad and Carney triad (CT). CSS is also known as the dyad of GIST and PGL; it affects both genders equally and is inherited in an autosomal dominant manner with incomplete penetrance. CT is a very rare disease; PGL, GIST and pulmonary chondromas constitute CT which shows female predilection and may be a mosaic disorder. Even though there is some overlap between CT and CSS, as both are due to *SDH* deficiency, CSS is caused by inactivating germline mutations in genes encoding for the SDH subunits, while CT is mostly caused by a specific pattern of methylation of the *SDHC* gene and may be due to germline mosaicism of the responsible genetic defect.

## Introduction

**Figure 1:**
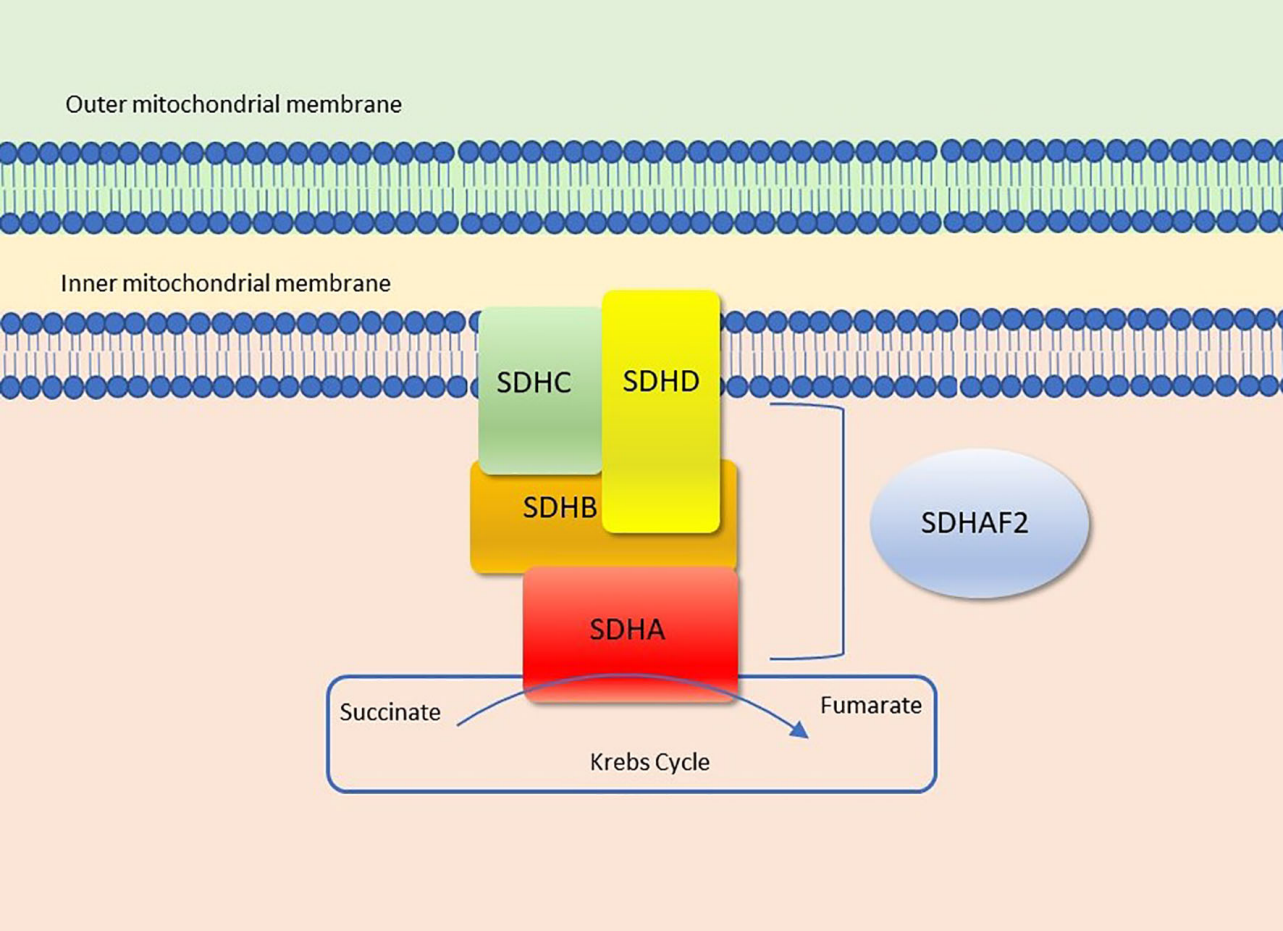
Succinate dehydrogenase (Complex II). Figure modified from Settas et al. (2).

Succinate dehydrogenase (SDH - also known as mitochondrial complex II or succinate-ubiquinone oxydoreductase) is the only enzyme that is concurrently both a functional member of both the Krebs cycle (or citric acid or tricarboxylic acid cycle) and the electron transport chain (ETC), where it provides electrons for oxidative phosphorylation (1). It is comprised of four mitochondrial subunit proteins: SDHA, SDHB, SDHC, SDHD encoded by nuclear genes, mapped to 5p15.22, 1p36.13, 1q23.3 and 11q23.1, respectively (**Figure 1**). SDHA is a flavoprotein and SDHB is an iron-sulfur protein; together they make up the main catalytic component of the complex. The other two subunits, SDHC and SDHD are two integral membrane proteins that anchor the complex to the inner mitochondrial membrane (3, 4). Additionally, the succinate dehydrogenase assembly factor 2 (SDHAF2) is required for the flavination and thus normal function of SDHA (4).

Genetic alterations in any of the four *SDHx* genes (*SDHA*, *SDHB*, *SDHC*, *SDHD*) or *SDHAF2* lead to SDH complex dysfunction and loss of SDHB expression (5). This loss of SDHB can be detected rapidly by immunohistochemistry (IHC) and thus, loss of immunohistochemical staining for SDHB is used as the hallmark of *SDH*-deficient tumors (6–9).

## How Do *SDHx* Mutations Lead to Tumorigenesis?

It is not completely clear how the dysfunction of SDH leads to neoplasia; several mechanisms have been proposed. One of them is the activation of pseudohypoxia pathway (10). This mechanism implies that due to *SDH* deficiency, succinate is accumulated; this inhibits propyl hydroxylases (PHDs) resulting in induction of the hypoxic response despite normoxic conditions (pseudohypoxia) (11, 12). At the cellular level, the three α subunits of the hypoxia inducible factor-1 (HIF-1α, HIF2α, HIF3α), are hydroxylated by PHDs 1, 2 and 3 (also known as *Egln2*, *Egln1* and *Egln3*), which are oxygen-dependent enzymes. The hydroxylated HIFαs are then targeted by von Hippel-Lindau (VHL) protein for degradation in the proteasome. In order for the HIFs to be recognized by the VHL, hydroxylation of two proline residues on HIFα is required by PHDs. In the case that the *SHDx* genes are mutated, propyl hydroxylases are inhibited by the accumulated succinate, hydroxylation of HIF-1αs is decreased and therefore they escape degradation. As a result, they translocate to the nucleus, they dimerize with HIFβ and create a complex that activates genes that induce angiogenesis, cell proliferation and glycolysis (12, 13). This mechanism was further supported by additional studies (12, 14–17). Additionally, besides succinate, the accumulation of reactive oxygen species (ROS) in mitochondria, leading to loss of function of the SDH enzyme, has also been implicated in tumor pathogenesis. ROS are mainly produced in complex I (NADH-ubiquinone oxidoreductase) and complex III (ubiquinone-cytochrome c oxidoreductase) in ETC (18). Recently, Xiao et al. demonstrate that *SDHx* knockdown increases intracellular levels of succinate; subsequently, this acts as an alpha-ketoglutarate competitor, inhibiting a-KG-dependent dioxygenases, JIp1, which is involved in sulfur metabolism and Jhd1 which belongs to the JmjC-domain-containing histone demethylase (JHDM) enzymes. That could lead to tumor formation by causing epigenetic changes (11, 19). The corresponding human JHDM, JMJD2D, was shown to be inhibited by accumulation of succinate as well (20).

Mutations in the *SDHx* subunits have been implicated in familial paragangliomas (PGLs) and pheochromocytomas (PHEOs), gastric stromal tumors (GISTs), Carney-Stratakis syndrome (CSS), and rarely in Carney triad (CT) and a few other tumors (21–30). This review focuses on *SDH*-deficient tumors, the two relevant genetic conditions, CSS and CT, and an association (3PAS), and their clinical, pathological and molecular characteristics.

## SDH-Deficient Paragangliomas and Pheochromocytomas

Pheochromocytomas and paragangliomas are rare neuroendocrine neoplasms derived from chromaffin cells (31). Tumors arising from the adrenal medulla, which is the largest paraganglion in the body, are termed PHEOs, while those derived from the sympathetic and parasympathetic paraganglia are known as PGLs (31). Extraadrenal locations most commonly include the head and neck, mainly the carotid body, jugular foramen, middle ear, but can also occur in the thorax, abdomen and pelvis (32). PGLs/PHEOs can be either sporadic or hereditary. As many as 35% of them are due to genetic predisposition (33). To date, more than 20 susceptibility genes have been identified (34). Germline mutations of *SDHB*, *SDHC*, and *SDHD* genes are responsible for approximately 50% of hereditary paragangliomas (4, 24, 25, 35–38) and pheochromocytomas (24, 36, 39). Recently, mutations in *SDHA* (21) and *SDHAF2* were also identified in hereditary PHEOs and PGLs (40). In addition, multiple reports have shown that these tumors have high incidence in patients with cyanotic congenital heart disease (41–43).

Depending on the *SDHx* subunit that is mutated, PGL syndromes have different characteristics (**Table 1**): *SDHD* (PGL1) (OMIM#168000)-mutated PGLs are more common in the head and neck and appear to have very high lifetime penetrance as 75% of carriers will have manifestations by 40 years old (44). Mutations in *SDHB* gene as the susceptibility gene for PGL4 (OMIM#115310) are more likely to be in the abdomen and show very high metastatic risk, but lower penetrance compared to PGL1 (~40% of carriers manifest the disease by age 40) (45). On the other hand, in *SDHC* (PGL3) (OMIM#605373) gene mutations, much rarer than the previous two, tumors are more commonly located in the carotid body (35, 46, 47) and have a low malignant potential (45). Mutations in *SDHA* and *SDHAF2* are associated with PGL5 (OMIM#614165) and PGL2 (OMIM#601650) respectively and are very rare. A patient with any type of PGL will present in any of the following contexts: a) because of signs and/or symptoms of excess catecholamine secretion (e.g. hypertension, headache, palpitations, hyperhidrosis, tremor); b) because of an incidental finding on an imaging study; c) because of signs and/or symptoms due to a local mass (various signs and/or symptoms depending on the location); and d) after a genetic testing was performed in the case of familial disease. Histologically, SDH-deficient PHEOs/PGLs have a nested architecture with round cells and prominent vasculature (4).

**Table 1:** Characteristics of SDH-deficient pheochromocytoma and paraganglioma.

Syndrome	Mutated gene	Mode of inheritance	Frequency	Maternal Imprinting	Affected gender	Associated tumors
**PGL1**	*SDHD* (11q23)	AD	Common	Yes	Both equally	Head and neck, intra-abdominal, adrenals, GIST
**PGL2**	*SDHAF2* (11q13)	AD	Very rare	Yes	Both equally	Head and neck
**PGL3**	*SDHC* (1q23)	AD	Rare	No	Both equally	Head and neck (carotid body), RCC
**PGL4**	*SDHB* (1p36)	AD	Common	No	Both equally	Intra-abdominal, head and neck, RCC
**PGL5**	*SDHA* (5p15)	AD	Rare	No	Both equally	GIST
**Carney triad**	Hypermethylation of *SDHC* promoter	Unknown	Very rare	No	Mainly females	GIST, abdomen, PCH

AD, autosomal dominant; GIST, gastrointestinal stromal tumor; PCH, pulmonary chondroma; PGL, paraganglioma; RCC, renal cell carcinoma.

PHEOs can occur as part of PGL1 and PGL4 and about 3% of them are attributed to *SDH* deficiency (6). The rest of them are either sporadic or they are associated with other familial syndromes such as VHL, MEN2 and NF. What could differentiate SDH-deficient PHEOs is the negative SDHB IHC and the secretion solely of noradrenaline (and/or dopamine) in contrast to the others that secret both adrenaline and noradrenaline (47). In addition, PHEOs caused by *SDHB* mutations show higher malignancy risk (47).

Family history is not always helpful in predicting hereditary PHEOs/PGLs because of phenotypic heterogeneity, incomplete penetrance and in the case of PGL1 and PGL2, maternal imprinting (5, 25, 48). It is interesting that in PHEOs/PGLs that appear to be sporadic based on family history, germline mutations were found in up to 25% of cases (49–51). Therefore, all patients with PHEOs/PGLs (sporadic and hereditary cases) should undergo genetic testing and counseling after IHC is performed (5, 6).

## SDH-Deficient GISTs

**Figure 2:**
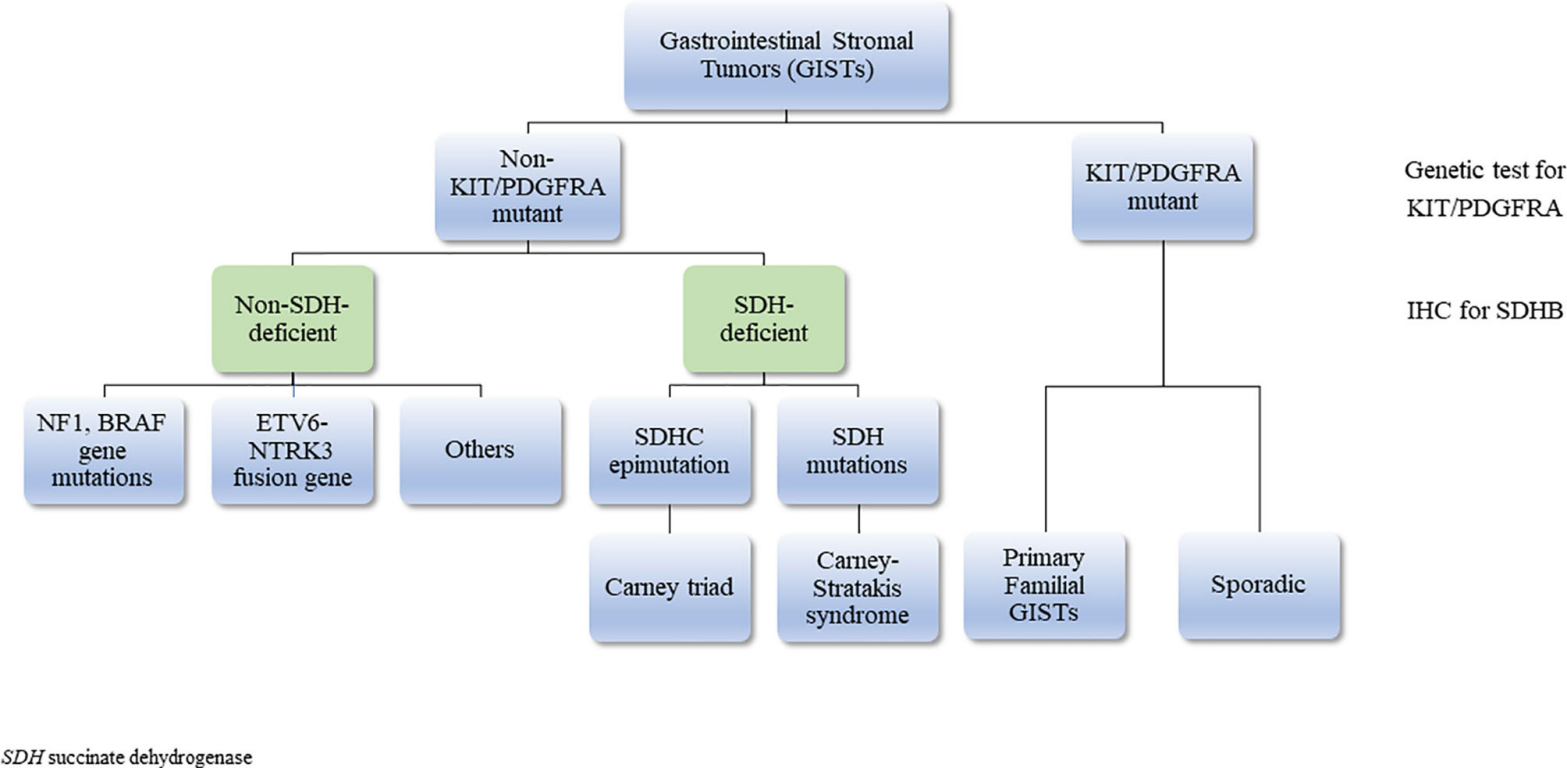
Classification of gastrointestinal stromal tumors (GISTs).

GISTs are the most common neoplasms of the gastrointestinal tract of mesenchymal origin and more than 5000 cases are diagnosed each year in the US alone (52). They originate from the interstitial cells of Cajal (53), the pacemaker cells that regulate peristalsis in the digestive tract (54). Most GISTs occurring in adults are driven by activating mutations in KIT proto-oncogene receptor tyrosine kinase (*KIT*) (75-80% of cases) or platelet-derived growth factor receptor A (*PDGFRA*) (5-15%) genes (55–58). The rest (10-15%), that lack *KIT* and *PDGFRA* gene mutations, are described as ‘wild type GISTs’ (WT GISTs) and comprise most of pediatric GISTs (59, 60). SDH-deficient GISTs are the majority of WT GISTs (50% of these tumors are associated with hypermethylation of the *SDHC* promoter locus (CT), 30% with germline *SDHA* mutations (4), while 20% is associated with mutations in *SDHB*, *SDHC*, *SDHD* (**Table 2**) (61). The rest harbor mutations in *NF-1, BRAF, ARID1A, ARID1B, CBL, NRAS, HRAS, KRAS, EGFR1, MAX, MEN1, PIK3CA* and *ETV6-NTRK3* fusion genes; these patients are usually older (same as KIT/PDGFRA + tumors) and they have more aggressive disease (62–72) (**Figure 2**). It is important to identify these mutations as it can be useful in the treatment plan.

**Table 2:** Comparison of SDH-deficient GISTs and SDH-competent GISTs.

	SDH-deficient GIST	Non-SHD deficient GIST
**Gender**	Female > male	Equal
**Age**	Children>young adult>older adult	Older adult
**Location**	Stomach	Anywhere in GIT
***KIT/PDGFRA* mutation**	No	Common (>90%)
***SDHB* IHC**	Positive	Negative
**Multifocality**	Rare	Common
**Predominant cell**	Spindled	Epithelioid
**Metastases to lymph node**	Common	Rare
**Response to imatinib**	No	Yes
**Associated syndromes/mutations**	50% *SDHC* epimutation (Carney triad) 30% germline *SDHA* mutation 20% *SDHB*, *SDHC*, *SDHD* mutation	Germline *KIT/PDGFRA* mutation, Neurofibromatosis 1 *BRAF, KRAS, NRAS, HRAS, ARID1A, ARID1B, CBL, FGFR1, MAX, MEN1, PIK3CA, ETV6-NTRK3*

GIST, gastrointestinal stromal tumor; GIT, gastrointestinal tract; IHC, immunohistochemistry; SDH, succinate dehydrogenase.

SDH- deficient GISTs exhibit unique features which are summarized in **Table 2**. Briefly, they manifest predominantly in females, at a young age. They arise almost exclusively in the stomach (61, 73–79) and they frequently have early lymphovascular invasion and consequent involvement of the lymph nodes (76), and less frequently of the liver (61), and do not frequently respond to imatinib (80). However, even in the setting of metastatic disease, they have an indolent clinical course. Histologically, these tumors exhibit multinodular growth pattern with epithelioid cells and they are multifocal. In addition, it was found that SDH-deficient GISTs overexpress insulin-like growth factor receptor (IGF1R) (81), and that this upregulation is highly specific of SDH-deficient GISTs (61, 78, 82, 83). The underlying molecular mechanism is unknown, but it could possibly be due to genetic amplification (61). Stratakis and his group also showed that immunohistochemistry that is negative for SDHB can be used to identify SDH-deficient GISTs caused by *SDHB*, *SDHC* or *SDHD* mutations (75). SDH-deficient GISTs can be sporadic or may present as part of two syndromes, CT (84) and CSS (26, 85).

### Carney Triad (CT)

Going back, in 1977 Dr. J. Aidan Carney described the association of three uncommon tumors- GISTs, PGLs and pulmonary chondroma (PCH) (86). Among other characteristics, the young age (median 18 years old), the female predilection, the multifocality and the concurrence of rare tumors suggested a genetic etiology (87). This association was later referred to as CT (OMIM #604287). Afterwards, adrenocortical adenoma and esophageal leiomyoma were added as components of the triad (88). The etiology of CT is not yet clear but recent data have implicated *SDHC*. In a cohort of 37 patients, comparative genomic hybridization demonstrated no mutations of any of the *SDHx* subunits. Instead, it revealed the most frequent and largest genomic change to be the deletion of 1q12-q21, a region where *SDHC* gene resides (84). Later, Haller et al. demonstrated that aberrant DNA hypermethylation is present at specific sequences of the *SDHC* gene (in the promoter and first exon) in patients with CT; this methylation leads to reduced *SDHC* mRNA expression (89). A genome-wide DNA study confirmed the *SDHC* gene promoter hypermethylation in both CT and WT-GISTs (90). Today, SDHC-specific methylation is considered the molecular signature of CT and is used as simple diagnostic test to identify lesions that may be part of CT in patients that are suspected to be affected by the condition.

### Carney-Stratakis Syndrome (CSS)

In 2002, Dr Carney and Dr Stratakis, described a new condition, that is today known as Carney-Stratakis syndrome (CSS) (OMIM #606864) (also reported as the “paraganglioma and gastric stromal sarcoma syndrome” or Carney-Stratakis dyad) (91). This newly described genetic disorder included only two types of tumors, PGLs/PHEOs and GISTs and is inherited in an autosomal dominant manner with incomplete penetrance. It affects both males and females during childhood and adolescence. Later, in 2007, Dr. Stratakis and his group identified inactivating mutations in the *SDHB*, *SDHC* and *SDHD* subunits as responsible for CSS (26, 92), with subunits B and D being mutated in higher frequency. Pasini et al. studied patients with CSS who developed GIST and they identified germline mutations in *SDHB*, *SDHC* and *SDHD* (26). Hemizygosity/homozygosity for the mutant allele was found in the GISTs of the affected individuals which is consistent with the tumor suppressor activity of *SDHx* genes (26). *SDHA* loss-of-function mutations have also been identified in patients with CSS (74). Surprisingly, patients harboring *SDHA* mutations demonstrated impressively long survival (93).

## 3PAS

Over the years, the co-existence of PHEOs/PGLs and pituitary adenomas (PAs) was thought to be a coincidence due to the rarity of those endocrine tumors (23). However, in some cases, they may have a common pathogenic mechanism. The first case of a patient with PHEO and acromegaly was described in 1952 (94). Since then, more than 80 such cases have been published (95). In 2012, Xekouki et al. described an individual within a family history with multiple PGLs and PHEOs caused by a germline *SDHD* mutation; in addition, the individual had an aggressive growth hormone (GH)-secreting PA, and loss of heterozygosity at the *SDHD* locus in the pituitary tumor along with increased levels of HIF-1α (96). Since then, the co-existence of those tumors, not recognized as a distinct entity before, has been known as 3PAs. More cases of PAs in patients with *SDH* mutations have been described, supporting the evidence that SDH deficiency plays a role in pituitary tumors (97–99).

SDH-deficient PAs that are part of 3PAs are more commonly macroadenomas and they frequently exhibit different phenotypes within the same family, such as prolactinomas, somatotropinomas and non-functional adenomas (95). Most of the time they respond poorly to somatostatin analogues and they require multiple treatments (95). In addition, PHEOs/PGLs in patients with 3PAs are often bilateral and/or multiple and tend to recur (95). In a cohort study of 19 patients with PHEO/PGL and PA, 9 of them had *SDHx* mutations. In PAs caused by mutations in any of the *SDHx* subunits intracytoplasmic vacuoles were present, a histological characteristic specific to those kinds of tumors (100). One could speculate that those vacuoles could possibly be autophagic bodies, as it is known that activation of autophagy is related to hypoxia-related pathways (101, 102); moreover, autophagy has been found to contribute to chemo- and radio-therapy resistance (103, 104).

## SDH-Deficient Renal Cell Carcinoma

SDH-deficient renal carcinoma was first recognized in 2004 (22) and later was accepted as a distinct type of renal cell carcinoma (RCC) (4, 105). It is rare, as it is estimated to account for 0.05-0.2% of all renal carcinomas (106). The mean age is 38 to 40 years (107) and there is a slight male predisposition (106, 108). In most of them, *SDHB* (83%) germline mutation is present (80), but few cases with *SDHC* and *SDHD* mutations have been reported as well (106–109). *SDHA* mutation in RCC was reported for the first time recently by Yakirevich et al. (110), followed by other reports (111, 112). In a cohort study, 36 SDH-deficient RCCs from 27 patients were studied; all of them were negative for SDHB and positive for SDHA by IHC. In addition, genetic testing was performed in 17 of these patients and they all harbored a germline *SDHx* mutation (16 SDHB, 1 SDHC) (106). In another study, 37 tumors exhibiting morphologic features of SDH-deficient RCC were evaluated; of them 11 showed immunohistochemical loss of SDHB and 1 out of 11 cases loss of SDHA (in this case no *SDHB* gene mutation was detected by sequencing and *SDHA* gene was not evaluated) (108).

Morphologically, SHD-deficient RCCs exhibit distinctive features, being made of cuboidal cells with variable cysts and ‘bubbly’ eosinophilic cytoplasm with flocculent inclusions. They also exhibit a solid, nested or tubular growth pattern (80, 106–108, 110, 113, 114). The hallmark of these tumors is loss of SDH immunohistochemical expression. Therefore, in renal tumors with morphology suggestive of SDH-deficient RCC or syndromic disease (younger age, family history of RCC, personal or family history of other SDH-deficient tumors) IHC for SDHB should be performed (106, 112). It is possible that SDHA-deficient RCCs may exhibit slightly different morphologic features such as papillary, cribiform-like architecture, higher nuclear grade and areas of solid growth pattern (110–112). However, very few cases have been reported so far and it is difficult to make any definitive associations.

In addition, this distinct type of RCCs is negative for c-kit, cytokeratin 7 (CK7), carbonic anhydrase IX (CAIX), CD117 and vimentin, while it is immunoreactive for PAX8 and kidney-specific cadherin. These markers can be useful in the case that IHC is unavailable (106–108).

Although most SHD-deficient RCCs have a good prognosis, and the risk of metastasis is estimated to be 11%, some of them- those with high-grade nuclear atypia, tumor necrosis or sarcomatoid differentiation- may behave aggressively reaching metastatic rates as high as 70% (106, 108, 115).

## Other SDH-Deficient Tumors

Apart from PGLs/PHEOs, GISTs, PAs and renal cell carcinomas discussed above, there is not much evidence that *SDHx* deficiency contributes significantly to other neoplasms. Thyroid carcinoma associated with either *SDHB* or *SDHD* has been reported in a few individuals (46, 51). Patients with PTEN-negative Cowden and Cowden-like syndromes, have also been reported in association with either *SDHB* or *SDHD* variants (27). Neuroblastoma (28) and bilateral adrenal medullary hyperplasia (29) have been linked to *SDHB* mutations. Moreover, a case of testicular seminoma has been reported in association with *SDHD* mutation (30). While a variety of tumors has been reported in association with SDH mutations we cannot say for sure if there is a causal relationship between them due to the very limited number of cases.

### Loss of SDHB Immunohistochemistry as an Important Tool of Validating SDH Mutations

*SDHx* genes act as tumor suppressor genes (116). *SDHx* germline heterozygous inactivating mutations affect the protein function and predispose to hereditary neoplasms; subsequently, loss of heterozygosity (LOH) in the tumor level results in complete loss of SDH activity (14). Loss of immunohistochemical staining for SDHB has been proved to be a robust and reliable marker for syndromic disease resulting from germline mutation of *SDHA*, *SDHB*, *SDHC* or *SDHD* (6–9). In addition, in the case of double-hit inactivation of *SDHA*, IHC for SDHA becomes negative as well (9, 21). Thus, tumors associated with bi-allelic inactivation of SDHA stain negative for SDHB and SDHA, while tumors caused by inactivating mutations in *SDHB*, *SDHC* or *SDHD* show negative staining only for *SDHB*. In every case, caution should be taken when interpreting the results and further clinical and genetic assessment should ensue.

## SDH-Deficient Tumors: Clinical Considerations and Genetic Counseling

Clinical features of the tumors discussed above should be taken into careful consideration. It is very important, in the case of PGLs/PHEOs, to be aware of any catecholamine excess symptoms (such as hypertension, hyperhidrosis, palpitations, headache) as well as signs and/or symptoms of a local mass. Depending on the tumor location they may vary. Tumors located in the carotid body may present with voice hoarseness, neck fullness, cough, dysphagia or clinically palpable mass in the lateral upper neck. When located in the middle ear (glomus tympanicum) patients may present with palsies of the cranial nerves VII, IX, X, XI and/or XII (117). It is recommended that these patients undergo imaging in order to detect metastatic disease or new tumors (118–120). In the case of *SDHA*, *SDHC* and *SDHD* mutations, because of the slow-growing tumors, MRI screening is suggested every three to five years (120). In individuals with *SDHB* mutations, due to the rapidly growing nature of these tumors, it should be performed every two years (120). A recent study demonstrated that the most optimal diagnostic imaging included MRI/CT and ^111^In-octreotide scintigraphy (121). Other studies showed higher sensitivity and more detailed imaging (regardless of genetic mutation and familial or sporadic cases) using ^68^Ga-DOTA-peptides PET/CT, which targets the abundantly expressed somatostatin receptors in those tumors, compared to conventional CT or MRI (122–128); in addition, more lesions were identified in the case of head and neck paragangliomas (HNPGLs) using that compared to all other imaging techniques (126) (including [^18^F]-fluorohydroyphenylalanine ([^18^F]-FDOPA) PET/CT, currently the gold standard for head and neck paragangliomas) (119, 129, 130). Patients with PA should be carefully examined for any symptoms of prolactin (PRL) or GH hypersecretion or visual disturbances, as most PAs that occur in the context of 3PAs are PRL- or GH- secreting macroadenomas or non-functional PAs. Complete pituitary hormone evaluation should also be performed to rule out other pituitary tumors. Hormonal testing should also be performed in the case of concurrent PGLs/PHEOs either in the index case or any family member. If there are no abnormal findings, based on the most recent recommendations, biochemical tests, including testing for PGLs/PHEOs, should be performed annually (118, 119). Pituitary MRI is indicated in the case of abnormal biochemistry or clinical findings. SDH-deficient PAs are treated the same as sporadic (131–134). In the case of renal cancer, patients may complain about flank pain and/or hematuria, whereas in GISTs abdominal pain or fullness may be the main issue.

### Genetic Counseling and Genetic Testing

It could be suggested that in the presence of SDH deficiency a careful and detailed medical and family history should be obtained even in patients with apparently ‘sporadic’ PGLs/PHEOs, GISTs or PAs due to the variable expression and decreased penetrance of those conditions. Patients and family members should be referred for genetic counseling. Genetic testing for *SDHx* mutations in any of the above patients, particularly if there are other family members with any of those tumors (do not only include first-degree relatives) should be performed. Doctors should be aware of CT or CSS especially in the case of a *KIT* or *PDFGRA* negative GIST. In the case that genetic testing is unavailable or cannot be performed, SDHB IHC could be performed.

## Summary

*SDH*-deficient tumors are often an indicator of a genetic, tumor-predisposition syndrome, associated with germline mutations in any of the *SDHx* subunits: *SDHA*, *SDHB*, *SDHC*, *SDHD* or rarely *SDHAF2*. In the case of CT, epimutation of *SDHC* promoter locus is the cause. Identifying the genetic basis of *SDH*-deficient tumors has helped in identifying individuals in high risk and introduce screening to them and their families. Thus, better clinical care can be provided as early detection and treatment have become more feasible.

## Author Contributions

GP contributed to writing the draft of the paper, to writing the final version of the paper, and critically revised it. FF and NS contributed to writing the final version of the paper and critically revised it. CS conceived the study and contributed to writing the final version of the paper and critically revised it. All authors contributed to the article and approved the submitted version.

## Funding

This study was supported by the Intramural Research Program, *Eunice Kennedy Shriver* National Institute of Child Health & Human Development (NICHD), Bethesda, Md 20892, USA.

## Conflict of Interest

CAS holds patent on the *PRKAR1A*, *PDE11A*, and *GPR101* genes and/or their function and his laboratory has received research funding from Pfizer Inc. FRF holds patent on the *GPR101* gene and/or its function. CAS is receiving compensation by ELPEN, Inc. Neither Pfizer, Inc nor ELPEN, Inc had any role in the study design, data collection and analysis, decision to publish, or preparation of the manuscript.

The remaining authors declare that the research was conducted in the absence of any commercial or financial relationships that could be construed as a potential conflict of interest.
